# A randomized double-blind control study of early intra-coronary autologous bone marrow cell infusion in acute myocardial infarction: the REGENERATE-AMI clinical trial^†^

**DOI:** 10.1093/eurheartj/ehv493

**Published:** 2015-09-24

**Authors:** Fizzah Choudry, Stephen Hamshere, Natalie Saunders, Jessry Veerapen, Katrine Bavnbek, Charles Knight, Denis Pellerin, Didier Locca, Mark Westwood, Roby Rakhit, Tom Crake, Jens Kastrup, Mahesh Parmar, Samir Agrawal, Daniel Jones, John Martin, Anthony Mathur

**Affiliations:** 1 Department of Cardiology, St Bartholomew's Hospital, Barts Health NHS Trust, West Smithfield, London EC1A 7BE, UK; 2 Stem Cell Laboratory, Barts Health NHS Trust and Blizard Institute, Queen Mary University of London, London, UK; 3 Institute of Cardiovascular Science, University College London, The Heart Hospital, UCLH, 16-18 Westmoreland Street, London W1G 8PH, UK; 4 Service de Cardiologie et Département de Médecine Interne, Centre Hospitalier Universitaire Vaudois, Lausanne, Switzerland; 5 Department of Cardiology, The Royal Free Hospital, Royal Free London Foundation Trust, London, UK; 6 Department of Cardiology, Rigshopitale, University of Copenhagen, Copenhagen, Denmark; 7 Cancer Division, Medical Research Council Clinical Trials Unit, London, UK; 8 British Heart Foundation Laboratories, Department of Medicine, University College London, London WC1E 6JJ, UK; 9 Barts Health NIHR Cardiovascular Biomedical Research Unit, Barts Health NHS Trust, London EC1A 7BE, UK

**Keywords:** Acute myocardial infarction, Stem cell therapy, Cardiac magnetic resonance imaging, Primary percutaneous coronary intervention

## Abstract

**Aims:**

Clinical trials suggest that intracoronary delivery of autologous bone marrow-derived cells (BMCs) 1–7 days post-acute myocardial infarction (AMI) may improve left ventricular (LV) function. Earlier time points have not been evaluated. We sought to determine the effect of intracoronary autologous BMC on LV function when delivered within 24 h of successful reperfusion therapy.

**Methods and results:**

A multi-centre phase II randomized, double-blind, and placebo-controlled trial. One hundred patients with anterior AMI and significant regional wall motion abnormality were randomized to receive either intracoronary infusion of BMC or placebo (1:1) within 24 h of successful primary percutaneous intervention (PPCI). The primary endpoint was the change in left ventricular ejection fraction (LVEF) between baseline and 1 year as determined by advanced cardiac imaging. At 1 year, although LVEF increased compared with baseline in both groups, the between-group difference favouring BMC was small (2.2%; 95% confidence interval, CI: −0.5 to 5.0; *P* = 0.10). However, there was a significantly greater myocardial salvage index in the BMC-treated group compared with placebo (0.1%; 95% CI: 0.0–0.20; *P* = 0.048). Major adverse events were rare in both treatment groups.

**Conclusion:**

The early infusion of intracoronary BMC following PPCI for patients with AMI and regional wall motion abnormality leads to a small non-significant improvement in LVEF when compared with placebo; however, it may play an important role in infarct remodelling and myocardial salvage.

**Clinical trial registration:**

Clinicaltrials.gov NCT00765453 and EudraCT 2007-002144-16.

**See page 264 for the editorial comment on this article (doi:10.1093/eurheartj/ehv541)**

## Introduction

Cardiovascular disease is the leading cause of death in the developed world, with global deaths due to coronary artery disease estimated to increase from 7.3 million in 2008 to 25 million by 2020.^1^ Despite significant improvement in outcome of acute myocardial infarction (AMI) with the introduction of primary percutaneous coronary intervention (PPCI), 30-day mortality is still estimated at ∼12%.^2^ While PPCI leads to reduced ischaemia, it is also associated with ischaemia-reperfusion injury which can account for up to 50% of final infarct size.^3^ Therefore, this improvement in survival may be associated with a relative increase in heart failure morbidity in patients with significant myocardial damage.

Preclinical studies have suggested that early delivery of bone marrow-derived cells (BMCs) following acute myocardial ischaemia results in reduction of infarct size.^4^ A number of phase I/II clinical trials assessing the safety and feasibility of autologous BMC delivery to the infarct zone after successful reperfusion of AMI have reported variable results^5–10^ and recent meta-analyses have demonstrated small improvements in left ventricular ejection fraction (LVEF).^11^ The optimal timing of cell delivery has not been established. Subgroup analysis of the REPAIR-AMI^12^ trial showed that the greatest benefit in LVEF was achieved with cell delivery at 5–7 days post-PPCI. The more recent TIME and LATE-TIME studies failed to show improvement in LVEF with cells delivered 3–7 days and 2–3 weeks post-AMI, respectively.^13,14^ Cells delivered at 24 h have also shown no improvement in LVEF.^9^ Concern regarding the safety of early cell delivery, 3–5 h post-reperfusion used in preclinical studies,^4^ has prevented investigation of an earlier time point in clinical studies. Assessment of ‘early’ cell delivery is essential in evaluating if cell therapy can be incorporated into the standard hospital care for patients with AMI.

REGENERATE-AMI is a multi-centre double-blind, randomized, and placebo-controlled trial designed to determine for the first time if early delivery of BMC following AMI is safe, efficacious in the improvement of left ventricular (LV) function and feasible, in the context of standard inpatient care for AMI.

## Methods

### Study design and participants

This is a multi-centre double-blinded, randomized, and placebo-controlled trial to determine if early intracoronary infusion of autologous bone marrow cells following AMI is safe, feasible and improves LVEF. The trial was approved by an independent ethics committee and registered at approved registries (ClinicalTrials.gov, NCT00765453 and EudraCT: 2007-002144-16). The full protocol has been previously published.^15^

Recruitment was from five centres (see Supplementary material online, Methods). Participants included in the trial had a diagnosis of acute anterior myocardial infarction (ST elevation in at least two contiguous anterior leads ≥0.2 mV), resultant significant anterior wall motion abnormality on LV angiography and had had successful PPCI within 24 h of symptom onset, defined as TIMI 3 flow in the infarct-related artery. Inclusion and exclusion criteria are detailed in Supplementary material online, Methods.

### Randomization and treatment

Following informed consent all participants underwent bone marrow harvest from the iliac crest under local anaesthesia as soon as possible after index PPCI (maximum time of 18 h allowing the recruitment of patients admitted out of hours). The aspirate was sent to the local designated stem cell laboratory where randomization occurred using dedicated trial software (IHD Clinical, Bishops Stortford, Herts, UK). Participants were randomized to either receive autologous bone marrow cells (BMC group) or matching placebo (placebo group) in a 1:1 block randomization allocation. Participants, investigators, and treating clinicians remained blinded to group assignment. The amount of bone marrow harvested was standardized at 100 mL. Bone marrow was processed as previously described.^16^ Briefly, BMC were isolated by density gradient centrifugation, washed and resuspended at a final volume of 10 mL. Placebo infusion was made up as 10 mL sterile NaCl 0.9% with 33 μL autologous whole bone marrow to match colour to the BMC product. No heparin was included in the final solution. The BMC/placebo product was infused into the infarct-related artery in three fractions during stop flow conditions within 6 h of bone marrow harvest.^12^

### Endpoints and definitions

The primary endpoint was absolute change in global LVEF assessed by advanced cardiac imaging at 1 year compared with baseline. Secondary imaging endpoints included changes in left ventricular volumes, area at risk (AAR) defined as myocardial oedema mass/LV mass and infarct size defined as infarcted LV mass/LV mass from baseline to 3 months and 1 year and change in global ejection fraction by LV angiography at 6 months. Other secondary endpoints included change in NT-proBNP levels (UK centres only), New York Heart Association (NYHA) classification, quality of life scoring (UK centres only), and major adverse cardiac events (MACE): all-cause mortality, recurrent AMI as per the universal definition of myocardial infarction,^17^ all coronary revascularisation (target vessel or non-target vessel). All adverse events were reported to 1 year and trial safety monitored by an independent Data and Safety Monitoring Board.

### Advanced cardiac imaging

Advanced cardiac imaging included cardiovascular magnetic resonance (CMR) or cardiac computed tomography (CT) for those unable to undergo CMR performed at 3 days post-PPCI (baseline), 3 months, and 1 year. Multi-phase cardiac data sets with full left ventricular coverage were acquired using standard protocols.^18^ The standard error of measurement of CMR and CT was 1.93 and 2.3%, respectively. The scans were anonymized, batched, and analysed (CVI42, Circle Cardiovascular Imaging Inc., Calgary, Canada) in a blinded fashion by two experienced operators. Briefly, each CMR examination used cine-CMR for ventricular volumes and function, T2-weighted imaging for myocardial oedema and delayed enhancement CMR for infarct size assessment. All cardiac CT analysis was performed using specialized cardiac CT circulation software with intraventricular tracings of end systolic and end diastolic timings. For full details of imaging protocols, see Supplementary material online, Methods.

### Left ventricular angiography

Participants underwent LV angiography and quantitative left ventricular (QLV) analysis at the time of index PPCI and at 6 months.

### Statistical design and analysis

The study was powered to the primary endpoint of change in LVEF at 1 year measured by CMR. The sample size was calculated using a two-sample *t*-test statistic to detect an improvement in LVEF of 6% with a power of 90% and significance level of 5%, based on previous cell therapy Phase I/II trials.^19^ The assumption was made that there would be little (<1%) improvement in the control group as previously shown.^19^ Accounting for an estimated drop-out of 25% at 1 year, we aimed to recruit a total of 100 participants to the study.

Analysis was based on the intention-to-treat principle. Baseline variables were summarized for each group. Continuous variables are presented as means ± SD or median ± interquartile range (IQR) and categorical variables are presented as percentages. 95% confidence intervals (CIs) are given. *P*-values are two sided with a value of <0.05 considered to indicate statistical significance. Within-group comparisons were performed using the paired *t*-test and repeated measures analysis of variance (ANOVA) adjusted for multiple comparison. Between-group comparisons were performed using the unpaired *t*-test. All statistical analyses were performed using SPSS version 19 (IBM Corp. Armonk, NY, USA) and graphs produced using Graphpad Prism version 5.0 (GraphPad Software, San Diego, CA, USA).

### Role of the funding source

No role of sponsor/funders in design or conduct of study.

## Results

**Figure 1:**
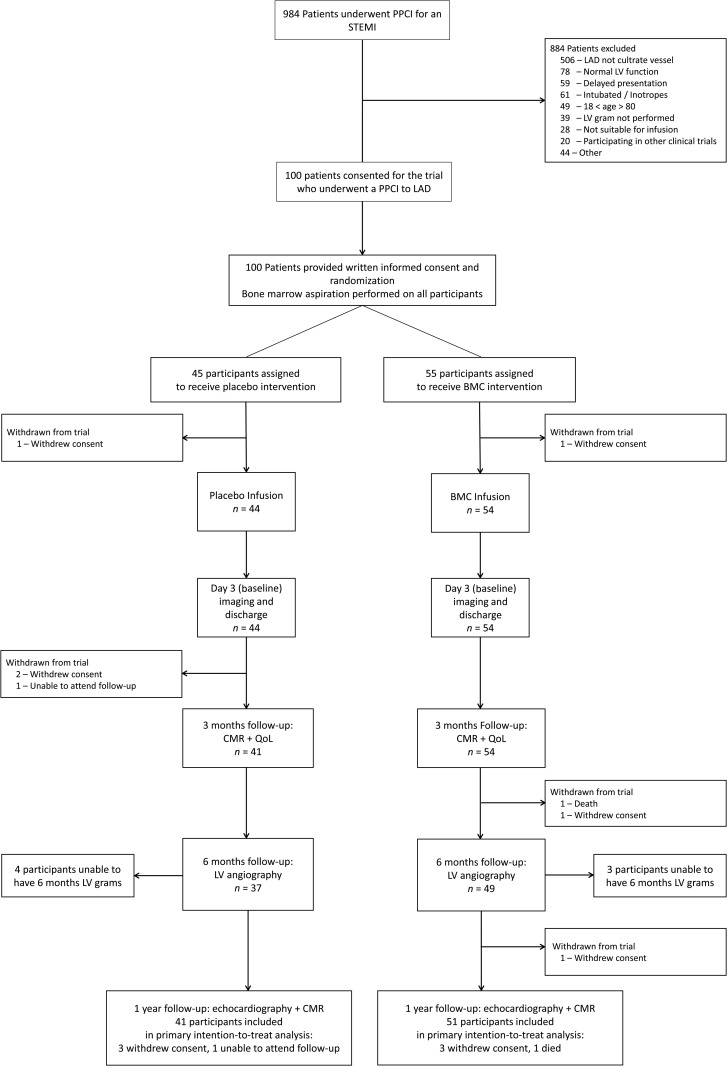
Consort diagram. Flow chart of the study design summarizing flow of participants through the trial.

Between 19 March 2008 and 5 March 2013, 984 patients with suspected ST elevation AMI were screened at five recruitment centres. Of these patients, 884 were excluded for: non-LAD culprit vessel (*n* = 506), no anterior wall motion abnormality on LV angiogram (*n* = 78), patient intubated/on inotropes (*n* = 61), delayed presentation (*n* = 59), age <18 or >80 (*n* = 49), and LV angiogram not performed (*n* = 39) (*Figure 1*). A total of 100 patients were recruited with 92 reaching the 1 year primary endpoint.

The mean age was 56.5 ± 10.5 years, and 87% were male. At baseline, the groups were similar with regards to age, sex, medical history, acute management, and findings on index angiography (*Table 1*). The median time from PPCI to intracoronary infusion was similar between the BMC group (532 min., range 403–1312) and the placebo group (583 min, range 458–1276), *P*-value: 0.74.

**Table 1:** Baseline characteristics of the study population

	Placebo group (*n* = 45)	BMC group (*n* = 55)	*P*-value
Age (year), mean ± SD	56.7 ± 10.7	56.4 ± 10.4	0.89
Sex (M/F), *n*)	41/4	46/9	0.27
Ethnicity (Caucasian) (*n*, %)	36 (80%)	47 (85.5%)	0.82
Medical history			
Hypertension (*n*, %)	12 (26.7%)	24 (43.6%)	0.080
Hypercholesterolaemia (*n*, %)	10 (22.2%)	19 (34.5%)	0.18
Diabetes mellitus (*n*, %)	4 (8.8%)	6 (10.9%)	0.74
Active smoker (*n*, %)	24 (53.3%)	27 (49.0%)	0.74
Previous MI (*n*, %)	1 (2.2%)	1 (1.8%)	0.89
Previous PCI (*n*, %)	0 (0%)	1 (1.8%)	0.37
Family history (*n*, %)	13 (28.8%)	17 (30.9%)	0.83
Medical therapy			
Aspirin (*n*, %)	45 (100%)	55 (100%)	1.0
Clopidogrel (*n*, %)	39 (86.7%)	50 (90.9%)	0.68
Prasugrel (*n*, %)	4 (8.9%)	3 (5.5%)	0.70
Ticagrelor (*n*, %)	2 (4.4%)	2 (3.6%)	1.0
Heparin (*n*, %)	40 (88.9%)	50 (90.9%)	0.75
Bivalirudin (*n*, %)	5 (11.1%)	5 (9.1%)	0.75
GP iib/iiia inhibitors (*n*, %)	33 (73%)	44 (80%)	0.63
DES used (*n*, %)	32 (71%)	36 (65%)	0.29
Concomitant PCI performed (*n*, %)	1 (2.2%)	3 (5.5%)	0.62
Baseline observations			
Blood pressure (diastolic/systolic), mean	138.6/85.6	138.0/83.9	0.89/0.62
Pulse (bpm), mean ± SD	84.5 ± 26.1	80.3 ± 19.8	0.44
BMI (kg/m^2^), mean ± SD	27.1 ± 4.3	26.7 ± 3.1	0.58
CCS >1 (*n*, %)	1 (2.2%)	5 (9.1%)	0.22
NYHA >I (*n*, %)	3 (6.7%)	4 (7.3%)	1.0
Angiographic findings			
BARI score (%)	35.6 (33.0–38.0)	35.6 (33.1–38.1)	0.98
APPROACH score (%)	37.1 (34.9–39.3)	38.5 (36.4–40.6)	0.37
TIMI flow <2 (*n*, %)	35 (77.8%)	38 (60%)	0.37
Timings			
Chest pain to PCI (min), median (IQR)	193.0 (145.5–320.5)	233 (155.0–348)	0.23
Door to PCI time (min), median (IQR)	36.0 (26.0–55.5)	40.0 (32.0–58.0)	0.22
PCI to BM aspiration time (min), median (IQR)	230.0 (112.0–966.0)	172.0 (105.0–976.0)	0.61
PCI to reinfusion (min), median (IQR)	583.0 (458.0–1276)	532.0 (403.0–1312)	0.74
BM aspiration to infusion (min), median (IQR)	313.0 (287.0–374.0)	323.0 (290.0–370.0)	0.41
Baseline LV function (CMR/CT)			
LVEF (%)	48.9 (45.9–51.9)	47.8 (45.2–50.3)	0.56
LVEDV (mL)	159.6 (149.5–169.7)	154.5 (145.5–163.4)	0.44
LVESV (mL)	81.4 (74.0–88.8)	81.8 (74.7–89.0)	0.93

Values are mean (95% CI).

BMI, body mass index (weight (kg)/height^2^ (m)); BM, bone marrow; BARI, Bypass Angioplasty Revascularization Investigation Myocardial Jeopardy Index; APPROACH, Alberta Provincial Project for Outcome Assessment in Coronary Heart Disease score; DES, drug eluting stent; LVEDV, left ventricular end diastolic volume; LVESV, left ventricular end systolic volume.

### Characterization of the cell therapy product

The total mononuclear cell count in the cell product was 59.8 × 10^6^ ± 59.9 with a mean percentage of viable cells of 97.6 ± 1.3 (Supplementary material online, *Table S1*). The mean number of CD34+ cells in the cell product (as a measure of stem-like potential of the BMC population) was 1.9 × 10^6^ ± 1.5. The placebo product contained 33 μL autologous whole bone marrow having an estimated mononuclear cell count of 20 × 10^3^ cells/mL and CD34+ cell count of 20 cells/mL.

### Left ventricular ejection fraction

**Figure 2:**
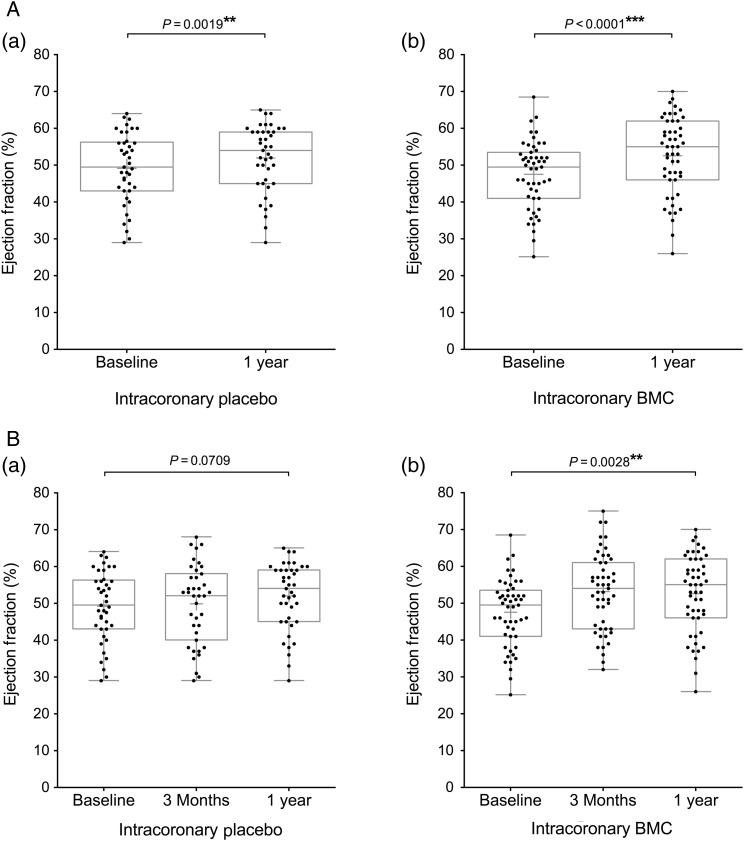
Endpoint analysis of left ventricular ejection fraction. Box and whisker plots (median and range, mean shown by +) including individual data points of left ventricular ejection fraction measured at (*A*) baseline and 1 year (significance measured by paired *t*-test); (*B*) baseline, 3 months, and 1 year (significance measured by repeated measures analysis of variance). Participant groups (a) intracoronary placebo infusion, (b) intracoronary BMC infusion.

Ninety-six participants underwent CMR assessment of cardiac function and two participants cardiac CT assessment. Baseline measurement of LVEF was similar in the BMC and placebo group (47.8 ± 9.4 and 48.9 ± 9.7%, respectively) (*Table 1*). Analysis of paired data revealed that global LVEF increased significantly in the BMC group by 5.1% from 47.5 ± 9.2% at baseline to 52.6 ± 10.5% at 1 year (*P* < 0.0001) and in the placebo group by 2.8% from 49.2 ± 9.6% at baseline to 52.0 ± 9.1% at 1 year (*P* = 0.0019) (*Figure 2*A). In a *post hoc* analysis, although there was a 2.2% (95% CI: −0.5 to 5.0; *P* = 0.10) absolute between-group difference in LVEF at 1 year favouring the BMC group, this did not reach statistical significance (Supplementary material online, *Table S2*).

Within-group changes at 3 months reveal a significant early increase in LVEF in the BMC group (5.7%, *P* = 0.0048) not seen in the placebo group (1.6%, *P* = 0.34). The significant change in LVEF at 3 months which is maintained to 1 year in the BMC group is reflected in the repeated measures ANOVA analysis where the overall change in LVEF is significant (*P* = 0.0028) compared with the placebo group (*P* = 0.071) (*Figure 2B*).

There were no within-group or between-group differences observed in LV end systolic volume, LV end diastolic volume or cardiac output at either 3 months or 1 year (Supplementary material online, *Table S2*).

### Infarct size, area at risk and myocardial salvage

Infarct size at Day 3 was smaller in the BMC group than in the placebo group (−5.4%; 95% CI: −9.4 to −1.4; *P* = 0.0084). There was a greater reduction in infarct size in the placebo group compared with the BMC group over time (4.1%; 95% CI: 0.3–7.9; *P* = 0.033). The AAR decreased from baseline to 1 year in both the BMC group by 32.5% (32.8 ± 9.6–0.3 ± 1.0%; *P* < 0.0001), and in the placebo group by 33.3% (34.3 ± 14.1–1.1 ± 3.5%; *P* < 0.0001) (Supplementary material online, *Table S2*).

Myocardial salvage index was calculated as the ratio of infarct size and AAR.^20^ At Day 3, the BMC group showed a greater myocardial salvage index compared with the placebo group (0.1%; 95% CI: 0.0–0.2; *P* = 0.048) (Supplementary material online, *Table S2*).

### Left ventricular function by left ventricular angiography

Global LVEF increased over 6 months in the BMC group by 7.1% (49.2 ± 11.1–56.4 ± 14.9%; *P* = 0.0007) and in the placebo group by 5.0% (52.4 ± 10.3–57.4 ± 12.1%; *P* = 0.012). There was a correlation between QLV LVEF and CMR LVEF in both groups (Supplementary material online, *Table S3* and Supplementary Data).

### Plasma NT-proBNP concentration

Plasma NT-proBNP was measured at baseline and 1 year and results underwent logarithmic transformation due to non-normal distribution. There was a similar within-group decrease in NT-proBNP at 1 year in the BMC group (1334 ± 1920–337.6 ± 615.2 pg/mL; *P* < 0.0001) and the placebo group (894.6 ± 994.7–214.3 ± 140.9 pg/mL; *P* = 0.0002) (Supplementary material online, *Table S4*).

### New York Heart Association

At 6 and 12 months >75% of participants had an NYHA I classification. There was no difference between NYHA classification between-groups.

### Quality of life

The European Quality of Life-5 Dimensions index score showed an improvement in symptoms in the placebo group over the first 6 months of 0.1 (0.7 ± 0.3 to 0.8 ± 0.3; *P* = 0.030). Within the BMC group, there was an improvement at 1 year, 0.1 (0.7 ± 0.4 to 0.8 ± 0.2; *P* = 0.040). The visual analogue scale showed similar improvement in both groups at 1 year (Supplementary material online, *Table S5*).

### Major adverse cardiac events and safety

Overall, there were very few MACE (Supplementary material online, *Table S6*) with a similar occurrence between-groups (*P* = 0.95) (Supplementary material online, *Figure S2*). There were no deaths in the placebo group whilst one patient died in the BMC group from myocardial infarction at 6 months. No cases of distal coronary artery occlusion occurred during intracoronary infusion; however, two participants underwent further coronary intervention at this time. Two participants had ventricular fibrillation between bone marrow harvest and infusion and were cardioverted successfully. Safety events are fully detailed in Supplementary material online, *Table S6*, importantly there are no significant between-group differences.

## Discussion

REGENERATE-AMI is the first randomized controlled trial to investigate if the early administration of BMC following revascularization for AMI, results in improved LVEF. Here the median time between PPCI and cell delivery was under 10 h, a time point closest to that used in successful preclinical work and previously not tested.^4^ REGENERATE-AMI demonstrates a significant improvement in LVEF at 1 year with a 5.1% increase in LVEF in the BMC group and a 2.8% increase in LVEF in the placebo group. Whilst these were significant within-group improvements, the between-group improvement favouring BMC was small and the trial therefore failed to achieve its primary endpoint. There was no between-group difference in secondary endpoints or safety endpoints. There was, however, a greater reduction in infarct size over 1 year in the placebo group compared with the BMC group.

Secondary endpoint analysis revealed a significantly smaller infarct size coupled with a significantly increased myocardial salvage index in the BMC group at Day 3 compared with the placebo group. These factors are known to be associated with AMI prognosis.^20^ As there was no significant difference in LVEF on angiography at PPCI, it could be suggested that the observed reduced infarct size and increased myocardial salvage were a result of BMC delivery. Interestingly, participants in the BMC group also had a higher angiographic risk score and a 40 min longer time from symptom onset to PPCI which should have resulted in a larger infarct size at the baseline scan. It is possible that intracoronary delivery of BMC at this early time point plays a role in limiting ischaemia-reperfusion injury which is estimated to account for up to 50% of final infarct size.^3^ Bone marrow mononuclear cells produce a variety of cytokines, chemokines, and growth factors that promote myocardial repair and their production is increased under conditions of hypoxic stress.^21^ It could therefore be hypothesized that secretion of these reparative paracrine factors is upregulated in the extensive myocardial hypoxia and inflammation present in the first 24 h following AMI.

The hypothesis that BMC promote early recovery of myocardial function is further supported by the early within BMC group improvement in LVEF at 3 months (47.5–53.3%, *P* = 0.0048) which was not seen in the placebo group. This difference favouring cell therapy is similar to the results seen at 6 months in the early trials upon which the assumptions were made for the design of REGENERATE-AMI.^19^ Although this early improvement in LVEF was maintained in the BMC group a later increase in LVEF in the placebo group meant that the difference between the two groups became small at 1 year, which has also been found in medium-term follow-up of previous trials.^6,7^ This as well as the fact that infarct remodelling is thought to be complete at 1 year provided the rationale to assess the primary endpoint for REGENERATE-AMI at 1 year.

Perhaps most importantly, REGENERATE-AMI shows that early infusion of stem cells in patients who have recently undergone PPCI (median—within 10 h) is safe. The potential of post-PPCI patients to become unstable within the first 24 h is well known.^22^ Therefore, cell delivery at this early stage may be limited by arrthymogenic risk of injecting into a hostile myocardium with extensive oedema, inflammation and microvascular obstruction, distal coronary embolization and reduction in coronary flow. In addition heavy antiplatelet and anticoagulant load may lead to bleeding. We showed a low rate of bleeding complications, no distal coronary occlusion, and the two participants who had ventricular arrhythmias were successfully cardioverted to sinus rhythm. The importance of assessing this time point was to be able to deliver cell therapy to patients following AMI within their standard 48 h hospital stay.^23^ We have shown that the delivery of BMC therapy is highly feasible within this timeframe without prolonging hospitalization. Cell therapy delivered at Days 3–7 or later might raise logistical concerns regarding its implementation into standard practice.

Despite evidence of positive effects of BMC therapy, REGENERATE-AMI failed to achieve its primary endpoint of an absolute improvement in LVEF of 6% in BMC-treated patients compared with placebo at 1 year. This was based on an early phase II trial and reflects the time at which REGENERATE-AMI was designed. A recent meta-analysis demonstrates a BMC conferred improvement in LVEF of 3.96%^11^ which may have been a more realistic estimation. A larger sample size may have allowed the difference to become significant but would have led to questions regarding the size of the effect and its clinical relevance. Another limitation of the trial is the relatively high ejection fractions seen at the baseline scan—this was a direct result of the examination of the early time point that meant that inclusion criteria were based on angiographic wall motion abnormality (i.e. some evidence of damage) and not 3D cross-sectional imaging which was not logistically possible if the aim was to reinfuse within 24 h. The trial therefore enrolled a relatively low proportion of participants with significantly impaired LVEF (<45%), a group shown to derive greatest benefit from BMC therapy.^11,12^ Cardiovascular magnetic resonance was performed at Day 3 post-infusion as this time point is considered to be representative of final infarct size.^24^ However, as there was no comparable imaging performed at the time of infusion it is not possible to conclusively attribute between-group differences in infarct size and myocardial salvage index at Day 3 to delivery of BMC or placebo.

REGENERATE-AMI uses an LVEF as a surrogate marker for the primary endpoint; however, the relationship between LVEF and hard clinical outcomes of mortality is not well defined. Furthermore, as both placebo and BMC groups had intracoronary injection, it might be hypothesized that improvement in LVEF in both groups was a result of ischaemic post-conditioning rather than a specific effect of the BMC itself. Whether there is ultimately an adjunctive benefit of BMC delivery in AMI will only definitively be addressed by a study that is powered to detect differences in mortality endpoints with a comparison with standard AMI care. The BAMI trial (BAMI, http://www.bami-fp7.eu) is the first Phase III trial designed to do this by enrolling 3000 participants with all-cause mortality at 5 years as the primary endpoint.

## Conclusion

REGENERATE-AMI demonstrates that intracoronary delivery of autologous BMC at an early time point (<24 h) after successful PPCI for anterior AMI leads to an insignificant improvement in LV function compared with placebo at 1 year. However, BMC therapy was associated with a reduction in infarct size and an increased myocardial salvage. Importantly, REGENERATE-AMI demonstrates the safety and feasibility of early intracoronary injection of BMC which supports the delivery of cell therapy within the timeframe of standard AMI hospitalization.

## Supplementary material

Supplementary material is available at *European Heart Journal* online.

## Funding

UK Stem Cells Foundation, the Heart Cells Foundation, and Barts and the London Charity. Funding to pay the Open Access publication charges for this article was provided by the Barts Cardiovascular Biomedical Research Unit (CVBRU).

**Conflict of interest:** none declared.

## Supplementary Material

Supplementary Data
