# Supplementary material for: A randomized double-blind control study of early intra-coronary autologous bone marrow cell infusion in acute myocardial infarction: the REGENERATE-AMI clinical trial
Source: Eur Heart J. 2015 Sep 24;37(3):256–63. doi: 10.1093/eurheartj/ehv493 (PMC4712349; doi:10.1093/eurheartj/ehv493)
Supplement: Supplementary Data [file ehv493_supplementary_data.zip › ehv493supp_data2.docx]

**Supplementary Results**

**Figure legends:**

**Figure S1:**

Relationship between baseline quantitate left ventricular left ventricular ejection fraction measured by left ventricular angiography and cardiac magnetic resonance left ventricular ejection fraction.

CMR – cardiac magnetic resonance; QLV – quantitate left ventricular angiography


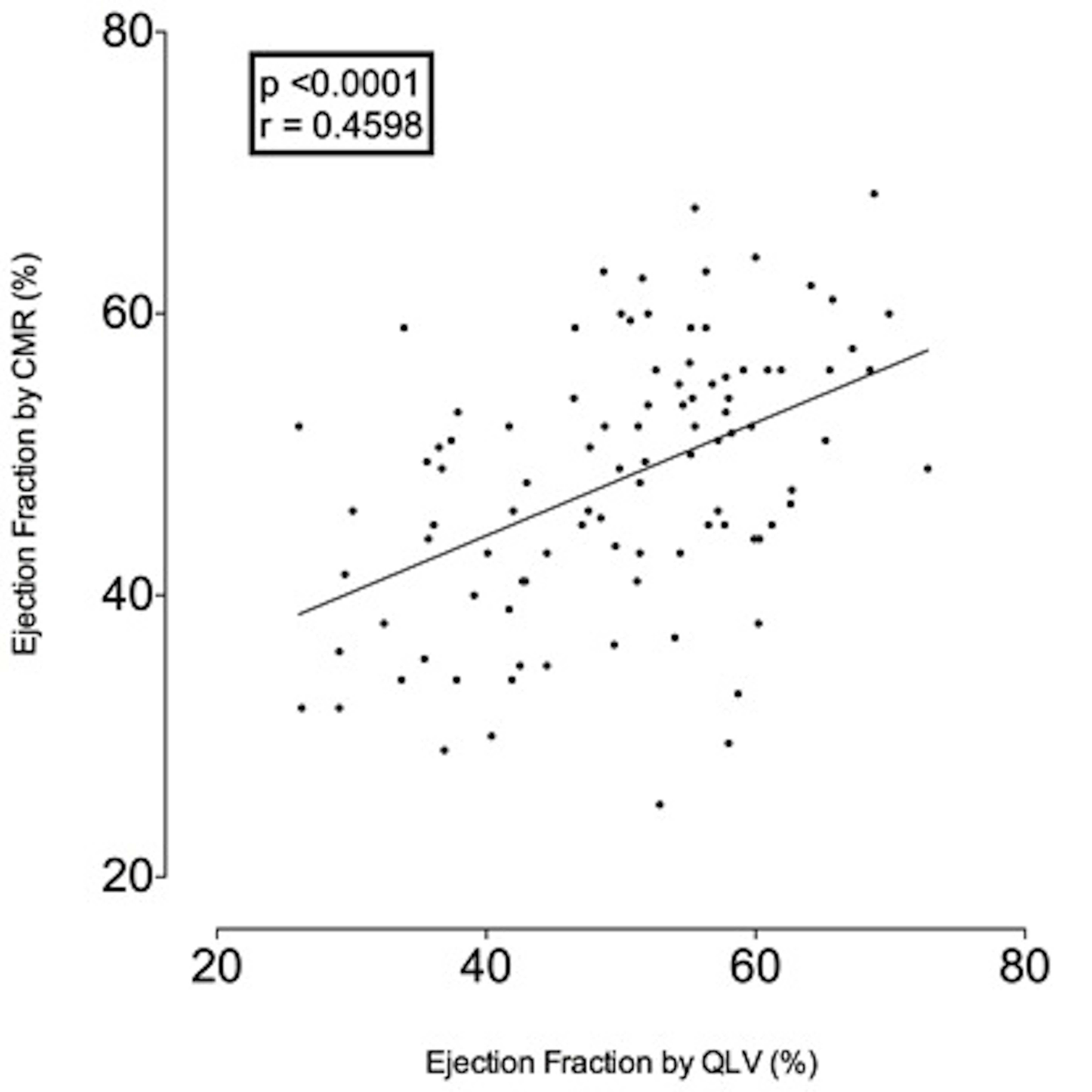


**Figure S2:**

Kaplan-Meier survival curves showing MACE during the follow-up visit


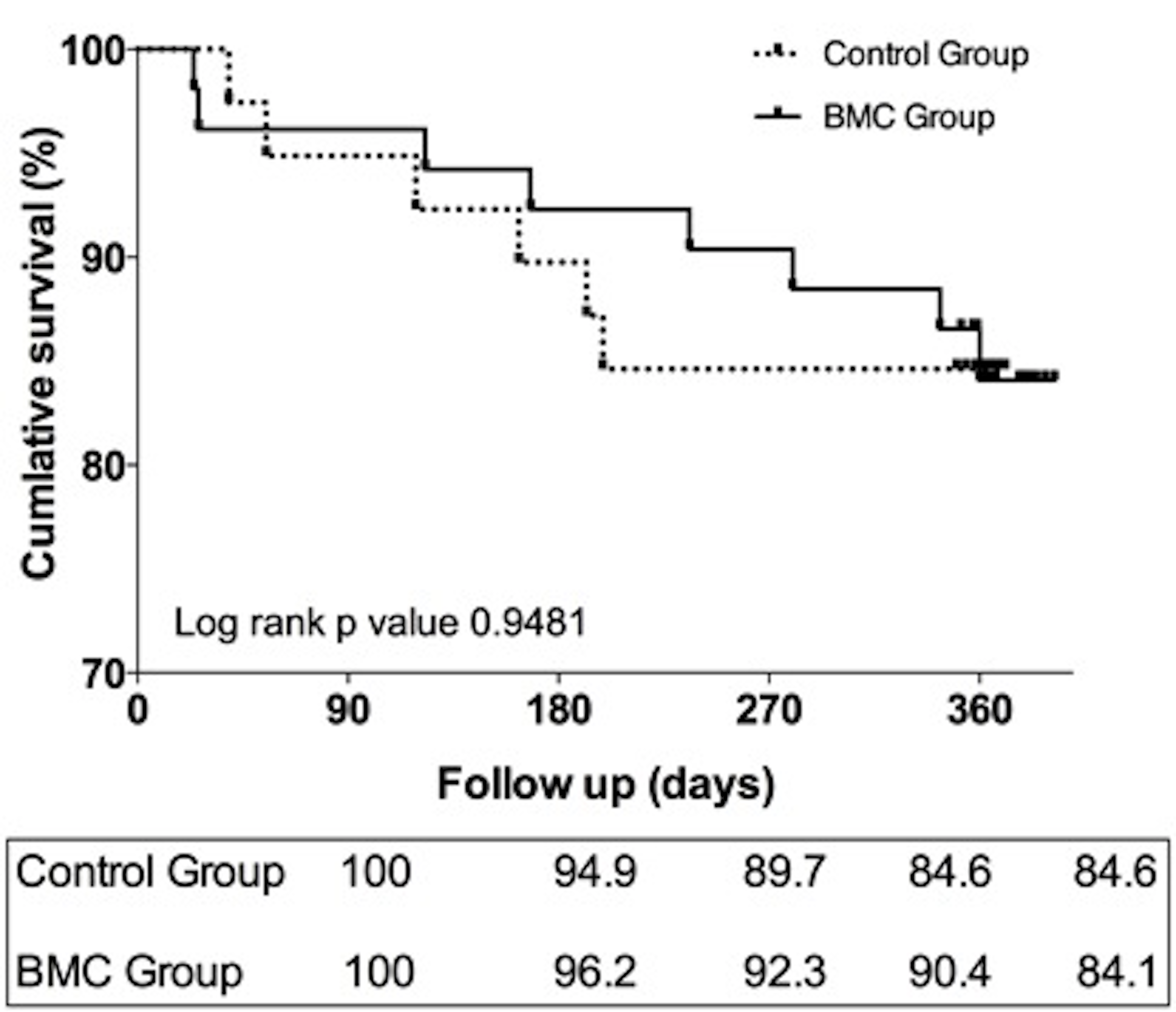


**Table S1: Infusion cell characteristics**

| Characteristic | n | Mean (95% CI) |
| --- | --- | --- |
|  |  |  |
| Number of mononucleated cells/product (x10^6^) | 91 | 59.8 (47.1 – 72.5) |
| Viability of cell product (%) | 91 | 97.6 (97.3 – 97.9) |
|  |  |  |
| CD34^+^ cells/product (x10^6^) | 91 | 1.9 (1.5 – 2.2) |
| EPC cells/product (x10^2^) | 48 | 75.2 (31.7 – 118.6) |
|  |  |  |
| Colony-forming units GM/1x10^5^ BMC | 29 | 114.3 (59.4 – 169.2) |
|  |  |  |

MNC – Mononuclear cells, CD34 - Hematopoietic progenitor cell antigen CD34, EPC - Endothelial progenitor cells expressing CD34^+^CD133^+^, GM - granulocyte-macrophage, BMC: Bone marrow cells

**Table S2. Primary endpoint and cardiac volumes**

|  | Placebo Group | BMC Group | Between group  P value |
| --- | --- | --- | --- |
| **Primary Endpoint** |  |  |  |
| 3 months paired samples | n = 40 | n= 53 |  |
| 1 year paired samples | n = 41 | n= 51 |  |
|  |  |  |  |
| LV ejection fraction |  |  |  |
| Baseline LVEF (%) | 49.2 (46.1 – 52.2) | 47.5 (44.9 – 50.1) | 0.41 |
| 3 months LVEF (%) | 49.9 (46.9 – 46.9) | 53.3 (50.4 – 56.2) |  |
| 1 year LVEF (%) | 52.0 (49.1 – 54.8) | 52.6 (49.6 – 55.5) |  |
|  |  |  |  |
| In group p value (3 months) | 0.34 | 0.0048 |  |
| In group p value (1 year) | 0.0019 | <0.0001 |  |
| RM ANOVA: In group p value  (baseline - 3months - 1 year) | 0.071 | 0.0028 |  |
|  |  |  |  |
| Difference in LVEF (%) |  |  |  |
| 3 months | 1.6 (-1.7 – 4.9) | 5.7 (2.6 – 8.8) | 0.082 |
| 1 year | 2.8 (1.1 – 4.5) | 5.1 (3.0 – 7.1) | 0.10 |
|  |  |  |  |
| **Secondary Endpoints** |  |  |  |
| 3 months paired samples | n = 40 | n= 53 |  |
| 1 year paired samples | n = 41 | n= 51 |  |
|  |  |  |  |
| LV End diastolic volume |  |  |  |
| Baseline LVEDV (mls) | 159.5 (148.9 – 170.0) | 155.2 (146.2 – 164.2) | 0.54 |
| 3 months LVEDV (mls) | 166.7 (149.9 – 170.2) | 162.6 (151.3 – 173.9) |  |
| 1 year LVEDV (mls) | 164.8 (153.6 – 176.1) | 164.2 (152.6 – 175.7) |  |
|  |  |  |  |
| In group p value (3 months) | 0.15 | 0.20 |  |
| In group p value (1 year) | 0.15 | 0.056 |  |
|  |  |  |  |
| Difference in LVEDV (mls) |  |  |  |
| 3 months | 6.6 (-2.4 – 15.7) | 7.1 (-2.3 – 16.5) | 0.95 |
| 1 year | 5.4 (-2.1 – 12.9) | 8.9 (-0.2 – 18.1) | 0.56 |
|  |  |  |  |
| LV End systolic volume |  |  |  |
| Baseline LVESV (mls) | 80.7 (73.3 – 88.0) | 82.5 (75.3 – 89.7) | 0.73 |
| 3 months LVESV (mls) | 84.3 (75.0 – 89.2) | 78.2 (69.3 – 87.1) |  |
| 1 year LVESV (mls) | 79.2 (71.2 – 87.3) | 80.0 (70.5 – 89.5) |  |
|  |  |  |  |
| In group p value (3 months) | 0.58 | 0.44 |  |
| In group p value (1 year) | 0.60 | 0.48 |  |
|  |  |  |  |
| Difference in LVESV (mls) |  |  |  |
| 3 months | 2.2 (-5.8 – 10.2) | -4.5 (-12.7 – 3.7) | 0.28 |
| 1 year | -1.4 (-6.9 – 4.1) | -2.5 (-9.5 – 4.5) | 0.82 |
|  |  |  |  |
| LV Cardiac Output (ml/min) |  |  |  |
| Baseline CO (ml/min) | 5076 (4608 - 5544) | 4655 (4272 – 5038) | 0.16 |
| 3 months CO (ml/min) | 5050 (4618 – 5483) | 4905 (4603 – 5207) |  |
| 1 year CO (ml/min) | 5179 (4789 – 5569) | 4879 (4528 – 5230) |  |
|  |  |  |  |
| In group p value (3 months) | 0.52 | 0.32 |  |
| In group p value (1 year) | 0.66 | 0.24 |  |
|  |  |  |  |
| Difference in CO (ml/min) |  |  |  |
| 3 months | -89.6 (-414.9 – -68.6) | 344.5 (-68.6 – 737.5) | 0.17 |
| 1 year | 103.3 (-382.5 – 589.1) | 224.0 (-155.5 – 603.4) | 0.69 |
|  |  |  |  |
| LV Myocardial Mass (g) |  |  |  |
| Baseline MM (g) | 119.3 (109.7 – 128.8) | 116.7 (109.7 – 123.8) | 0.66 |
| 3 months MM (g) | 106.3 (97.81 – 114.7) | 100.5 (92.93 – 108.1) |  |
| 1 year MM (g) | 107.8 (98.89 – 116.6) | 96.86 (89.39 – 104.3) |  |
|  |  |  |  |
| In group p value (3 months) | 0.0041 | 0.0002 |  |
| In group p value (1 year) | 0.0011 | <0.0001 |  |
|  |  |  |  |
| Difference in MM (g) |  |  |  |
| 3 months | -12.2 (-20.3 – -4.1) | -15.7 (-22.5 – 8.9) | 0.52 |
| 1 year | -11.5 (-18.1 – 4.8) | -19.9 (-25.9 – -13.8) | 0.063 |
|  |  |  |  |
| 3 months paired samples | n=35 | n=50 |  |
| 1 year paired samples | n=37 | n=45 |  |
|  |  |  |  |
| LV Infarct size (%) |  |  |  |
| Baseline IS (%) | 20.1 (16.6 – 23.7) | 14.7 (12.4 – 17.0) | 0.0084 |
| 3 months IS (%) | 13.4 (10.2 – 16.6) | 10.4 (7.7 – 13.1) |  |
| 1 year IS (%) | 11.4 (8.3 – 14.4) | 10.1 (7.1 – 13.1) |  |
|  |  |  |  |
| In group p value (3 months) | 0.0004 | 0.0006 |  |
| In group p value (1 year) | <0.0001 | 0.0003 |  |
|  |  |  |  |
| Difference in IS (%) |  |  |  |
| 3 months | -6.6 (-10.0 – -3.2) | -4.8 (-7.0 – -2.7) | 0.35 |
| 1 year | -8.8 (-11.9– -5.7) | -4.7 (-7.1 – -2.3) | 0.033 |
|  |  |  |  |
| 3 months paired samples | n=30 | n=41 |  |
| 1 year paired samples | n=33 | n=41 |  |
|  |  |  |  |
| LV Area at Risk (%) |  |  |  |
| Baseline AAR (%) | 34.3 (29.5 – 39.2) | 32.8 (29.8 – 35.9) | 0.79 |
| 3 months AAR (%) | 4.1 (2.0 – 6.2) | 4.42 (2.2 – 6.7) |  |
| 1 year AAR (%) | 1.1 (0.0 – 2.3) | 0.3 (0.0 – 0.6) |  |
|  |  |  |  |
| In group p value (3 months) | <0.0001 | <0.0001 |  |
| In group p value (1 year) | <0.0001 | <0.0001 |  |
|  |  |  |  |
| Difference in AAR (%) |  |  |  |
| 3 months | -31.4 (-36.3 – -26.4) | -27.8 (-31.2 – -24.4) | 0.22 |
| 1 year | -33.2 (-38.9 – -27.4) | -32.5 (-35.5 – -29.5) | 0.83 |
|  |  |  |  |
|  | n=37 | n=45 |  |
|  |  |  |  |
| Myocardial Salvage Index |  |  |  |
| MSI | 0.4 (0.4 – 0.5) | 0.5 (0.5 – 0.6) | 0.048 |
|  |  |  |  |

Values are mean (95% CI). LVEF - left ventricular ejection fraction, LVEDV - left ventricular end diastolic volume, LVESV - left ventricular end systolic volume, LVSV - left ventricular stroke volume, MO – Myocardial oedema, CO – Cardiac Output

**Table S3:** **QLV endpoint and cardiac volumes**

|  | Placebo Group | BMC Group | Between Group |
| --- | --- | --- | --- |
| 6 months paired samples | (n = 39) | (n= 48) | p value |
| **LVEF (%)** |  |  |  |
| Baseline LVEF (%) | 52.4 (49.1 – 55.8) | 49.2 (46.0 – 52.5) | 0.22 |
| 6 months LVEF (%) | 57.4 (53.5 – 61.4) | 56.4 (52.0 – 60.7) |  |
|  |  |  |  |
| In group p value (6 months) | 0.0121 | 0.0007 |  |
|  |  |  |  |
| Difference in LVEF (%) |  |  |  |
| 6 months | 5.0 (1.2 – 8.9) | 7.1 (3.2 – 11.1) | 0.45 |
|  |  |  |  |
| **LVEDV (mls)** |  |  |  |
| Baseline LVEDV (mls) | 142.6 (130.3 – 154.8) | 130.0 (117.1 – 143.0) | 0.096 |
| 6 months LVEDV (mls) | 149.8 (137.5 – 162.2) | 142.9 (130.8 – 155.0) |  |
|  |  |  |  |
| In group p value (6 months) | 0.13 | 0.037 |  |
|  |  |  |  |
| Difference in LVEDV (mls) |  |  |  |
| 6 months | 7.3 (-2.2 – 16.7) | 12.8 (1.0 – 24.6) | 0.47 |
|  |  |  |  |
| **LVESV (mls)** |  |  |  |
| Baseline LVESV (mls) | 68.1 (60.5 – 75.7) | 67.2 (59.1 – 75.3) | 0.61 |
| 6 months LVESV (mls) | 56.6 (56.6 – 71.3) | 62.5 (52.6 – 72.4) |  |
|  |  |  |  |
| In group p value (6 months) | 0.21 | 0.32 |  |
|  |  |  |  |
| Difference in LVESV (mls) |  |  |  |
| 6 months | -4.1 (-10.7 – 2.4) | -4.7 (-14.1 – 4.8) | 0.93 |
|  |  |  |  |
|  |  |  |  |

Values are mean (95% CI). LVEF - left ventricular ejection fraction, LVEDV - left ventricular end diastolic volume, LVESV - left ventricular end systolic volume, LVSV - left ventricular stroke volume

**Table S4: NT-proBNP Results**

|  | Placebo Group | BMC Group | Between Group |
| --- | --- | --- | --- |
|  |  |  | P value |
| 1 year paired samples | (n= 32) | (n= 44) |  |
| **Log NT-proBNP Results** |  |  |  |
| Baseline | 2.6 (2.4 – 2.8) | 2.8 (2.6 – 2.9) | 0.30 |
| 6 months | 2.2 (2.1 – 2.4) | 2.3 (2.1 – 2.5) |  |
| 1 year | 2.2 (2.1 – 2.3) | 2.2 (2.0 – 2.3) |  |
|  |  |  |  |
| In group p value (6 month) | 0.0051 | 0.0005 |  |
| In group p value (1 year) | 0.0002 | <0.0001 |  |
|  |  |  |  |
| Difference at 6 months | -0.3 (-0.5 – -0.1) | -0.4 (-0.6 – -0.2) | 0.58 |
| Difference at 1 year | -0.4 (-0.6 – - 0.2) | -0.6 (-0.8 – -0.4) | 0.33 |
|  |  |  |  |

Values are mean (95% CI). NT-proBNP - N-terminal prohormone brain natri­uretic peptide

**Table S5: Quality of life (EQ5D)**

| 1 year paired samples | Placebo Group  (n=25) | BMC Group  (n=30) | Between Group  P value |
| --- | --- | --- | --- |
| **EQ5D** |  |  |  |
| Index Score – Baseline | 0.7 (0.5 – 0.8) | 0.7 (0.5 – 0.8) | 0.46 |
| Index Score – 6 months | 0.8 (0.7 – 0.9) | 0.7 (0.6 – 0.9) |  |
| Index Score – 12 months | 0.8 (0.6 – 0.9) | 0.8 (0.7 – 0.9) |  |
|  |  |  |  |
| In group p value (6 months) | 0.030 | 0.11 |  |
| In group p value (1 year) | 0.17 | 0.040 |  |
|  |  |  |  |
| Difference in Index Score |  |  |  |
| 6 months | 0.1 (0.0 – 0.2) | 0.1 (-0.0 – 0.2) | 0.92 |
| 1 year | 0.1 (-0.0 – 0.2) | 0.1 (0.0 – 0.3) | 0.75 |
|  |  |  |  |
|  |  |  |  |
| VAS – Baseline | 59.4 (48.1 – 70.6) | 66.5 (59.0 – 74.1) | 0.68 |
| VAS – 6 months | 65.0 (54.0 – 76.4) | 71.8 (62.7 – 80.8) |  |
| VAS – 12 months | 71.6 (63.5 – 80.4) | 73.8 (66.0 – 81.5) |  |
|  |  |  |  |
| In group p value (6 months) | 0.19 | 0.0070 |  |
| In group p value (1 year) | 0.033 | 0.046 |  |
|  |  |  |  |
| Difference in VAS |  |  |  |
| 6 months | 9.4 (-5.0 – 23.8) | 9.0 (2.7 – 15.3) | 0.95 |
| 1 year | 12.6 (1.2 – 24.1) | 7.3 (0.1 – 14.4) | 0.39 |
|  |  |  |  |

Values are mean (95% CI). EQ5D - European Quality of Life-5 Dimensions VAS – Visual analogue scale

| **Safety events (AE, SAE, MACE) at 1 year** | **Placebo group** | **BMC group** | **p-value** |
| --- | --- | --- | --- |
|  | **(n=45)** | **(n=55)** |  |
|  |  |  |  |
| **Total no. of events** | 31 | 41 | 0.13 |
| **Participant, No. (%)** | 20 (44.4) | 28 (50.9) | 0.55 |
| **Total MACE** | 7 | 12 | 0.19 |
| **Participant, No. (%)** | 7 (15.6) | 8 (14.5) | 1.0 |
| **Breakdown of MACE:** |  |  |  |
| All cause mortality | 0 | 1 | 1.0 |
| Recurrent AMI | 2 | 3 | 1.0 |
| Target vessel revascularisation | 3 | 7 | 0.50 |
| Non-target vessel revascularisation | 2 | 0 | 0.20 |
| ICD insertion | 0 | 1 | 1.0 |
| **Total AE/SAEs** | 24 | 29 | 0.44 |
| **Breakdown of AE/SAEs:** |  |  |  |
| VF arrest post BM aspiration | 2 | 0 | 0.20 |
| Post-procedural minor bleeding | 2 | 1 | 1.0 |
| Non-clinically significant contamination of bone marrow aspirate | 2 | 3 | 1.0 |
| Hepatitis C +ve | 1 | 0 | 1.0 |
| HIV +ve | 0 | 1 | 1.0 |
| Admission with Troponin -ve chest pain | 11 | 9 | 1.0 |
| LV thrombus on imaging (Echo/CMR) | 2 | 5 | 0.45 |
| Post-AMI pericardial effusion | 0 | 1 | 1.0 |
| Post-procedural renal impairment | 0 | 1 | 1.0 |
| Ablation for atrial fibrillation | 0 | 1 | 1.0 |
| Non-cardiac cause for hospital admission | 3 | 6 | 0.51 |
| Drug reaction requiring hospital admission | 1 | 1 | 1.0 |
|  |  |  |  |

**Table S6: All documented adverse events at 1 year**
