# Supplementary material for: A randomized double-blind control study of early intra-coronary autologous bone marrow cell infusion in acute myocardial infarction: the REGENERATE-AMI clinical trial
Source: Eur Heart J. 2015 Sep 24;37(3):256–63. doi: 10.1093/eurheartj/ehv493 (PMC4712349; doi:10.1093/eurheartj/ehv493)
Supplement: Supplementary Data [file ehv493_supplementary_data.zip › ehv493supp_data1.docx]

**Supplementary methods**

**Participants**

Patients were recruited from 5 centres; The London Chest Hospital (London, UK), Royal Free Hospital (London, UK), Heart Hospital (London, UK), Centre Hospitalier Universitaire Vaudois (Lausanne, Switerzland) and Rigshospitalet (Copenhagen, Denmark).

**Inclusion Criteria:**

Patients have to fulfil the following criteria:

1. Patients presenting with acute anterior myocardial infarction (ST elevation in at least 2 contiguous anterior leads ≥0.2 mV) and treated with acute percutaneous coronary intervention (PCI) with stent implantation within 24 h after symptom onset.
2. Acute PCI/stent implantation has been successful (residual stenosis visually <30% and TIMI flow ≥2).
3. At the time of inclusion the patient no longer requires intravenous catecholamines or mechanical hemodynamic support (aortic balloon pump).
4. Significant regional wall motion abnormality on left ventricular angiogram in left anterior descending (LAD) territory at the time of acute PCI.
5. Age 18–80 years (primary angioplasty confers an adverse prognosis in those over the age of 80 years).
6. Written informed consent.

**Exclusion Criteria:**

Patients are excluded if one of the following criteria is met:

1. Regional wall motion abnormality not consistent with culprit vessel.
2. Need to revascularise additional vessels, outside the infarct artery as a planned procedure (these vessels can be treated at baseline).
3. Arteriovenous malformations or aneurysms.
4. Active infection, or fever or diarrhoea within the past 4 weeks.
5. Chronic inflammatory disease.
6. Known HIV infection or active hepatitis.
7. Neoplastic disease without documented remission within the past 5 years.
8. Cerebrovascular insult within 3 months.
9. Impaired renal function (creatinine >200 mmol) at the time of cell therapy.
10. Significant liver disease (gamma-glutamyltrasferase >2× upper limit) or spontaneous International Normalised Ratio >1.5).
11. Anemia (haemoglobin <8.5 mg/dL).
12. Platelet count <100 000/µL.
13. Hypersplenism.
14. Known allergy or intolerance to clopidogrel, heparin or abciximab.
15. History of bleeding disorder.
16. Gastrointestinal bleeding within 3 months.
17. Major surgical procedure or trauma within 2 months.
18. Uncontrolled hypertension.
19. Pregnancy.
20. Mental retardation leading to inability to obtain informed consent.
21. Previously performed stem/progenitor cell therapy.
22. Participation in another clinical trial within the past 30 days.

**Advanced Imaging Protocols**

**CMR Protocol**

CMR imaging was performed on a local 1·5T scanner with a cardiac 32-channel phased array coil. Each examination used cine-CMR for ventricular volumes and function, T2-weighted imaging for myocardial oedema and delayed enhancement CMR for infarct size assessment and evaluation of microvascular obstruction (MVO). Cine CMR is considered the gold standard of the evaluation of cardiac volumes, mass, and systolic function since it does not apply geometric assumptions and has excellent reproducibility and accuracy (standard error for left ventricular, LV, mass and volume is approximately 5%) (Grothues et al., 2004; Hundley et al., 2010). Balanced steady-state free precession cine imaging was used to acquire 10­12 short axis slices (8 mm slice thickness, 2mm gap) with one slice per breath-hold. Sequence parameters were 1.5 ms echo time (TE), 3.1 ms repetition time (TR), and acquired voxel size was 1.8 x 1.86mm with a typical field of view (FOV) of 360mm in the phase encode direction. 45 phases were acquired with 25% phase sharing. Parallel imaging (SENSE) was used with an acceleration factor of 2.0.

Delayed gadolinium enhancement (DE) images are T1-weighted inversion recovery sequences acquired about 10 minutes after the intravenous administration of gadolinium with an inversion time chosen to null the myocardial signal (Simonetti et al., 2001) using an ‘inversion time scout’ or ‘look locker’ sequence. Gadolinium is an extracellular agent, which enhances its distribution volume in certain conditions such as necrotic myocardium, assuming a bright signal (hyperenhancement), opposed to dark viable (normal) myocardium (Judd et al., 1995). CMR highlights the region of scar as small as 0.16 g (Wu et al., 2001) and the reproducibility is high with a coefficient of reproducibility reported equal to+2.4% of LV mass in the chronic setting (Mahrholdt et al., 2002). Delayed enhancement images were acquired ten minutes after injection of a dose of 0.2 mmol/kg of gadoterate meglumine (Dotarem) for delayed gadolinium enhancement. A T1-weighted segmented inversion-recovery gradient echo pulse sequence (TR 3.9ms TE 1.9ms, flip angle 15^o^, voxel size of 2 x 2mm, typical FOV 360mm) was used to obtain 10­12 short axis slices (matched with short-axis cine images) with one slice per breath-hold. The inversion time was adjusted individually according to a T1 scout sequence (Look-Locker). Images were acquired every other heart beat with 2 signal averages.

Myocardial oedema is one of the earliest manifestations of ischaemia and occurs before the development of definitive and irreversible damage, T2-weighted imaging may be used to visualise the ischaemic area at risk (AAR) (Beek and van Rossum, 2010). Increased myocardial water content increases signal on T2-weighted images due to a prolongation of the T2 relaxation time (Higgins et al., 1983). Myocardial oedema in the acute phase of AMI can therefore be visualized as a bright signal on T2-weighted images, defining the ‘myocardium or area at risk’ (Aletras et al., 2006). Myocardial oedema was assessed using fat suppressed T2-weighted triple inversion turbo spin echo STIR (Short tau inversion recovery) imaging (TE 80ms, TR 2 heart beats, TSE factor 31, voxel size 1.8 x 1.8mm). 10-12 slices were obtained (8mm per slice, 2mm gap matched to DE/cine slices) with one slice per breath-hold.

Images were anonymised, batched and analyzed in blinded fashion by two experienced operators. Scar and oedema volumes were calculated by manually drawing endocardial and epicardial contours followed by semi-automated selection of normal remote myocardium per slice. Myocardial oedema was described as >2SD in signal intensity from remote normal myocardium. Infarct size was calculated using the full-width half maximum method as previously described (Flett et al., 2011). In case of discordance between operators, blinded review by a level III accredited CMR reader was performed (Dr Mark Westwood). Analysis was performed using dedicated software (CVI^42^, Circle Cardiovascular Imaging Inc, Calgary, Alberta, Canada).

**Cardiac CT Protocol**

All patients were studied on a CG-gated 64-slice spiral CT (Sensation 64, Siemens, Forchheim, Germany) scanner. Intravenous B-Blockers were used in patients to achieve optimal pulse rate prior to initiation of the scan, all patients were on cardiac monitors during administration. All scans were performed in the craniocaudal direction. An initial scouting x-ray (topogram) is performed to ensure correct alignment of the patient for the remainder of the scan. The scan is initiated when contrast arrives within the ascending aorta, this can be performed manually after test bolos or using an automated CT triggering once the Hounsfield unit crosses a threshold in a set region of the aorta. A total of 50 - 100 mls of contrast is used for the scan with a duration of 10 – 15 minutes for the scan. Reconstruction algorithms were used to convert the raw data into interpretable images which is then analyzed on specialist software.

**Left ventricular volumes and function – analysis**

The data is reconstructed throughout the cardiac cycle and cine movie images are collected in 10 phases along the cardiac cycle and 10 levels. Analysis of LV volumes and function was performed using dedicated PC-based software Siemens (Forchheim, Germany) is used to calculate epicardial and endocardial borders in both systole and diastole

**Left ventricular angiography**

The LV angiogram was performed in the 30^o^ RAO position and recorded at a minimum of 15-30 frames per second, contrast was injected over 10 seconds. Quantitative left ventricular analysis (QLV) was performed using the centreline method using QAngioXA 7.3 (Medis)(Assmus et al., 2002).
